# The influence of television on the food habits of schoolchildren and its association with dental caries

**DOI:** 10.1002/cre2.244

**Published:** 2019-09-05

**Authors:** Regina de Nazaré Marreiros Tavares Silva, Danilo Antonio Duarte, Arlete Maria Gomes de Oliveira

**Affiliations:** ^1^ Department of Pediatric Dentistry Clinic Aplicação School of Federal University of Pará—UFPA Belém Pará Brazil; ^2^ Department of Pediatric Dentistry Cruzeiro do Sul University São Paulo Brazil; ^3^ Department of Public Health São Leopoldo Mandic School of Dentistry Campinas Brazil

**Keywords:** child, dental caries, food habits, television

## Abstract

**Objectives:**

The consumption of food with a high‐sugar content is encouraged by the food industry through television (TV) aimed at children and may be associated with dental caries. This study aims to evaluate the influence of TV on the food habits of schoolchildren aged years and its association with dental caries.

**Material and methods:**

This was an observational, epidemiological, and cross‐sectional study. Five neighborhoods of Belem District were selected, and then two schools from each neighborhood were drawn (one private and one public). All sixth and seventh grade students were selected. Data were extracted from questionnaires completed by schoolchildren and their parents and the decayed, missing, and filled teeth (DMFT/dmft) indices of the schoolchildren. The indices were carried out by three examiners previously calibrated (κ > .80). Logistic regression analysis was performed to investigate the association of variables of study with consumption of cariogenic foods and occurrence of dental caries. Odds ratios (ORs) and 95% confidence intervals (CIs) were calculated.

**Results:**

Schoolchildren who watched TV for >90 min were more likely to consume cariogenic foods (OR = 2.38; 95% CI [1.57, 3.60]) and have a DMFT + dmft >1 (OR = 2.10; 95% CI [1.37, 3.26]). Those who consumed cariogenic foods while watching TV were more likely to have DMFT + dmft >1 (OR = 14.75; 95% CI [8.24, 6.40]). Parents who bought foods they saw on TV contributed to a higher consumption of cariogenic foods (OR = 3.29; 95% CI [2.07, 5.24]) and DMFT + dmft >1 (OR = 3.93; 95% CI [2.09, 7.37]) among their children.

**Conclusions:**

TV can influence the eating habits of schoolchildren aged 10 to 12 and the food purchases of their parents, stimulating the consumption of cariogenic foods and contributing to the development of dental caries.

## 1 INTRODUCTION

Eating habits are built from birth and transformed throughout life by the interaction of environmental, nutritional, social, economic, psychological, and cultural factors(Köster, 2009). Globally, television (TV)—still considered the most used form of media for marketing food and beverages, especially those with high levels of sugars, fats, and salt—plays an influential role in food choices(Cairns, Angus, Hastings, & Caraher, 2013). Thus, exposure time to TV can be considered a risk factor for the higher consumption of foods and beverages of low nutritional quality through the influence of advertisements on food preferences(Boyland et al., 2011) and the consumption of unhealthy snacks in front of the TV, especially when the public comprises children and adolescents(Oliveira et al., 2016).

A high‐sugar diet is an important risk factor not only for obesity, diabetes, and other chronic noncommunicable diseases (CNCDs) but also for dental caries, which is considered the most prevalent CNCD and has the second‐highest incidence in the world(Mennella, 2014; Vos et al., 2017). This issue has a large impact on the budget of health care in low‐income countries and reaches approximately 60–90% of the world's population of school‐age children(Treerutkuarkul & Gruber, 2015). As a result, the World Health Organization (WHO) has established a new guideline for the prevention of these chronic diseases by reducing the consumption of free sugars by adults and children to less than 10% of the total caloric intake and, in a way that could lead to an even greater reduction, to less than 5% of the total caloric intake(World Health Organization, 2015).

However, sugar consumption has been stimulated by the secondary sector of the economy through the production of ultraprocessed foods and beverages, which are considered unhealthy; through globalized marketing strategies; and through information and communication technologies used to stimulate the purchase of these products(Pan American Health Organization, 2012).

The relationship between TV and inadequate eating habits is already well established, especially when it refers to the effective influence of marketing on the eating behaviors of children and adolescents, which contributes to an increase in the rates of overweight and obesity in these age groups(Boyland & Halford, 2013; Jenkin, Madhvani, Signal, & Bowers, 2014; Lobstein & Dibb, 2005). However, the association of this relationship with dental caries is still unclear.

Therefore, considering that children and adolescents watch TV for an average of 2 hr daily(Oliveira et al., 2016) and that dental caries is associated with the frequent presence of free sugars (carbohydrates) metabolized by the biofilm disbiotic microbiota, leading to enamel demineralization(Sheiham & James, 2015) and presenting a cumulative effect throughout life(Broadbent, Foster Page, Thomson, & Poulton, 2013), this research aimed to analyze the influence of TV on the eating habits of schoolchildren from 10 to 12 years of age and their possible association with dental caries. We realized that schoolchildren that were watching more TV were more likely to consume cariogenic foods and have dental caries. The control of factors that contribute to the occurrence of caries in childhood would have a positive impact on disease indices in adulthood, thus contributing to the development of adequate oral health and indirectly reducing the risk factors common to other CNCDs.

## 2 MATERIALS AND METHODS

### 2.1 Study design and ethical approval

This was an observational, epidemiological, cross‐sectional study that was approved by the Research Ethics Committee of the São Leopoldo Mandic Dental School (opinion No. 1,922,660/CAAE 64287617.5.0000.5374); the study was conducted between March 2017 and January 2018 in the city of Belém, Pará, Brazil. The results of the last epidemiological survey on oral health, SB Brazil 2010, showed that the northern region, where the state of Pará is located, had the lowest percentage (28%) of 12‐year‐olds free of caries(Brasil. Project SB Brazil, 2010). The city of Belém, the 12th‐most populous municipality in Brazil, presented a mean decayed, missing, and filled teeth (DMFT) of 2.45 for individuals aged 12 years, with 87.7% of the index represented by the worst condition of the disease—caries lesion and total tooth loss(Brasil. Project SB Brazil, 2010).

This study was conducted according to the Screening the Reporting of Observational Studies in Epidemiology(Malta, Cardoso, Bastos, & Magnanini, 2010) statement and according to the principles defined in the Declaration of Helsinki.

### 2.2 Sample size calculation and sampling of schoolchildren

The sample size was calculated according to the parameters of the National Oral Health Survey—SB Brazil 2010: a proportion of 72% of 12‐year‐old individuals affected by caries disease in northern Brazil, a confidence level of 95%, a power of 80%, a standard error of 5%, and a design effect 2, plus a 10% nonresponder rate. The sample size calculated for this study was 612 students. Sampling was carried out by clusters in two stages (neighborhoods and schools). The BioEstat® 5.3 (Civil Society Mamirauá, Manaus, Amazonas, Brazil) statistical program was used to randomly select five of the nine neighborhoods of the administrative district of Belém as primary units. For each neighborhood, two schools (one public and one private) were randomly selected, giving a total of 10 schools as secondary sampling units. All students in the sixth and seventh grades of elementary school among the selected schools were included in the survey, which resulted in the initial sample of 681 students who received the informed consent form and self‐administered questionnaire to complete at home with their parents and who were asked to return it 7 days following delivery. The questionnaire comprised 14 closed and two open‐ended questions addressed to the students and three closed questions addressed to the parents. The questionnaire was adapted from two questionnaires—the Ghimire and Rao Questionnaire(Ghimire & Rao, 2013), translated into Portuguese, and the National School Health Survey Questionnaire (PENSE)(*Instituto Brasileiro de Geografia e Estatística (IBGE): Pesquisa nacional de saúde do escolar*, 2015). The reliability of the instrument was previously obtained during a pilot study by test and retest (Cronbach's *α* = .80).

### 2.3 Criteria for the inclusion and exclusion of schoolchildren

The initial inclusion criteria were students regularly enrolled in the sixth and seventh grades of elementary school among the selected schools who were cognitively able to answer the questionnaire and who presented the free and informed consent form signed by them and the parents. Of the 681 schoolchildren in the initial sample, 136 schoolchildren were excluded: 52 returned the completed questionnaire incorrectly, 28 were submitted to diet control by nutritionists, and 56 refused participation. There was no replacement of sample elements. The sample resulted in 545 schoolchildren.

The students were submitted to oral clinical examination in the school environment, performed under natural light using a mouth mirror (Golgran, São Paulo, SP, Brazil) and CPI probe (Trinity, São Paulo, SP, Brazil), according to WHO guidelines for the epidemiological survey of dental caries disease(World Health Organization (WHO), 2013). The exam was carried out by three examiners previously calibrated in a pilot study (*κ* > .80) using the DMFT index for permanent teeth and dmft index for deciduous teeth. The calibration process was performed according to the WHO guidelines of the methodological basis for oral health research(World Health Organization (WHO), 2013). The first step involved a theoretical discussion on the diagnosis of caries. The second step involved the oral clinical examination of 15 children for the determination of interexaminer agreement.

However, 35 schoolchildren were absent on the day of the epidemiological survey of caries. The final sample resulted in 510 students aged 10 to 12 years and their parents.

### 2.4 Sampling and inclusion/exclusion criteria for advertisements

Then, the school calendar was obtained to allow for the drawing of the month and the first day of recording the programming of the two most assisted channels, as indicated by the students in the questionnaire. Recordings occurred during the school period (15 consecutive days in November 2017) and during the vacation period (15 consecutive days in January 2018) from 8 a.m. to 10 p.m., totalling 420 hr of recording. An external hard drive with a 500‐Gb storage capacity (HX‐MU050DA/AA2—Samsung Electronics—Manaus, Brazil) and a 22‐inch LCD TV (Model L22 W831—AOC—Envision Industry, Brazil) were used for the recordings of each channel (Electronic Products, Brazil).

All commercial food and beverage advertisements shown during the schedule intervals of the two selected channels during the 30‐day recording were included in the analysis, excluding noncommercial advertisements, such as those with political content.

### 2.5 Classification of advertisements

The food and beverage advertisements were identified, analyzed, and classified according to the following: the content (product marketed); the frequency of presentation; the food pyramid group(Philippi, Latterza, Cruz, & Ribeiro, 1999); the type of processing used in the production, based on the Dietary Guidelines for the Brazilian Population (in terms of natural, culinary ingredients and processed and ultraprocessed ingredients)(Brasil, 2014); and the sugar content, based on the criteria of the Food Standards Agency, the regulatory agency of the United Kingdom, categorized as low content (<2 g/100 g), medium content (2–9 g/100 g), and high content (>10 g/100 g)(Morgan, Fairchild, Phillips, Stewart, & Hunter, 2009).

### 2.6 Dependent variables (outcomes), independent variables, and covariables

Two outcomes were considered: “caries experience” and “consumption of cariogenic foods while watching TV.”

The caries experience of schoolchildren was obtained by means of an epidemiological survey of caries performed using the dmft and DMFT indices. The values of the indices were summed to assign the total burden of disease in each individual of the sample. Similarly, all index components (decayed, missing due to caries, and filled teeth) were considered because the M/m (missing teeth) component represents the most severe stage of the disease and the F/f (filled teeth) component represents the disease stage in the past, because the restored tooth has already been a previously decayed tooth.

Food products consumed by schoolchildren while watching TV and reported in the questionnaire were listed and classified as “potentially cariogenic foods/beverages” and “noncariogenic foods/beverages” according to the sugar content added to the food, as indicated in the Table of Nutritional Composition of Food Consumption in Brazil(Instituto Brasileiro de Geografia e Estatística (IBGE)., n.d.), and according to the product labels and the Food Standards Agency criteria. Processed and ultraprocessed foods and beverages were considered to be potentially cariogenic, with high‐free sugar content (above 10 g/100 g) added in their formulations. Foods that did not present free sugars in their composition or that presented low‐sugar content (<2 g/100 g) were considered noncariogenic foods(Brasil, 2014; World Health Organization, 2015).

The main independent variables were related to TV viewing habits and consumption habits. They were all obtained through the following items on the questionnaire: “TV time,” “time spent in front of TV,” “watching TV commercials,” “buy food you see on TV,” “buy beverages you see on TV,” and “buy oral hygiene products you see on TV.”

The covariates referred to age, sex, nature of the school environment (public or private), visit to the dentist, and type of consultation performed. Age was obtained in years and categorized into two ranges: 10–11 years and above 11–12 years. The variable “type of consultation performed” was categorized as unrealized, preventive (all caries disease prevention procedures), and treatment (all procedures for caries lesion repair). The type of school (public or private) was used as a proxy for socioeconomic status.

Data on the variables related to the parents (i.e., “child asks for foods that he or she see on TV,” “parents buy requested foods,” and “parents buy food that they watch on TV”) were collected through the questionnaire for the parents through four levels of answers, two of which were affirmative (always and almost always) and two of which were negative (rarely and never).

The variables related to the advertisements (“frequency of presentation,” “recording period,” and “sugar content of product”) were collected through the recordings of channels.

### 2.7 Data analysis

The data collected from the self‐administered questionnaire were tabulated in an Excel spreadsheet (Microsoft® Excel 2007 software package—Microsoft Corporation, Redmont, WA, USA), and after the descriptive analysis of the data by means, medians, standard deviations, frequencies, and percentages, simple logistic regression models were fitted for each analyzed variable, determining their associations with the outcomes “cariogenic food consumption while watching television” and “caries experience” (dmft + DMFT). DMFT and dmft caries indices were summed and dichotomized by the median (=1) for the association analyzes. This is done when applying logistic regression, so that the categories are balanced and the power of the test is higher.

The strength of the associations was analyzed with brute odds ratios and 95% confidence intervals. The variables with *p* < .20 in the crude analyses were tested in multiple logistic regression models, remaining as models with *p* ≤ .05. Multiple associations were analyzed by adjusted odds ratios and 95% confidence intervals. The analyses were performed in the program R (Statistical Package R Core Team (2017); R: A language and environment for statistical computing, R Foundation for Statistical Computing, Vienna, Austria; URL HTTPS: http://www.R-project.org/). For this type of analysis, it was taken into consideration that the objective of this study was to evaluate the eating habits of children and adolescents when staying in front of the TV, its influence on food choices for consumption, and its possible association with dental caries. Therefore, the variability among participants was more important because, despite being grouped in schools, watching TV and diet are usually habits developed individually by students in their homes, under the influence of family. For this reason, we considered in the analysis the variables of individuals. This was decided on the basis of the current situation, concluding that with the access to social networks and the great mobility of children between the schools within the same category, for our purpose, there was no reason to think that children from the same school from the same city and perhaps even from other cities show less variability among themselves than when compared with children from other schools of the same category (public or private), regarding these associations analyzed.

For the purposes of statistical analysis and the calculation of mean values of caries indices for the deciduous teeth of schoolchildren, the numerical values of the DMFT equivalent index were used (A = 0, B = 1, C = 2, D = 3, E = 4, F = 5, G = 6, H = 7, K = 8, T = T, and L = 9). Advertising data were placed in an Excel spreadsheet for descriptive analysis and chi‐squared (χ^2^) tests.

## 3 RESULTS

A total of 510 students with a mean age of 11.2 years (±0.84) were evaluated (55.8% female and 54.4% students from private schools). The mean value for dmft + DMFT was 1.74 (±1.81). Concerning the TV‐watching habits, 98% of schoolchildren watched TV channel programming and 2% watched NETFLIX (global film provider) content on TV, with TV viewing at night being more frequent. The association of the variables with the “caries experience” outcome is shown in Table 1. This association was positive for the following variables: the time spent watching TV, the purchase of products viewed on TV by schoolchildren and their parents, and the consumption of potentially cariogenic foods while watching TV. The associations between the studied variables and the “consumption of cariogenic foods while watching television” outcome are shown in Table 2.

**Table 1:** Association of variables with caries experience of Brazilian schoolchildren, 2017

Variable		*n* (%)	dmft + DMFT		OR brute (CI 95%)	*p* value	OR adjusted (CI 95%)	*p* value
			<1 *n* (%)	≥1 *n* (%)				
Age (year)	>11ª	243 (47.6)	128 (52.7)	115 (47.3)				
	≤11	267 (52.4)	134 (50.2)	133 (49.8)	1.10 [0.78, 1.56]	.5747		
Gender	Femaleª	285 (55.9)	149 (52.3)	136 (47.7)				
	Male	225 (44.1)	113 (50.2)	112 (49.8)	1.09 [0.76, 1.54]	.6442		
School	Privateª	278 (54.5)	144 (51.8)	134 (45.2)				
	Public	232 (45.5)	118 (50.9)	114 (49.1)	1.04 [0.73, 1.47]	.8331		
TV time	Morningª	193 (37.1)	103 (63.4)	90 (46.6)		.4785		
	Night	317 (61.0)	159 (50.2)	158 (49.8)	1.14 [0.80, 1.63]			
Time spent watching TV (min)	<90ª	289 (56.7)	180 (62.3)	109 (37.7)				
	>90	221 (43.3)	82 (37.1)	139 (52.9)	2.80 [1.95, 4.02]	<.0001	2.10 [1.37, 3.26]	.0008
Watch TV commercials	Nª	388 (74.6)	204 (52.6)	184 (47.4)				
	Y	122 (23.5)	58 (47.5)	64 (52.5)	1.22 [0.81, 1.84]	.3450		
Buy food you see on TV	Nª	283 (54.4)	171 (60.4)	112 (39.6)				
	Y	227 (43.6)	91 (40.1)	136 (59.9)	2.28 [1.60, 3.26]	<.0001	1.87 [1.21, 2.90]	.0047
Buy soft drinks you see on TV	Nª	455 (87.5)	246 (54.1)	209 (45.9)				
	Y	55 (10.6)	16 (29.1)	39 (70.9)	2.87 [1.56, 5.28]	.0039		
Buy oral hygiene products you see on TV	Nª	491 (94.4)	256 (52.1)	235 (47.9)				
	Y	19 (3.6)	6 (31.6)	13 (68.4)	2.36 [0.88, 6.31]	.1408		
Visit to the dentist	N	159 (31.2)	72 (45.3)	87 (54.7)	1.43 [0.98, 2.08]	.0645		
	Yª	351 (68.8)	190 (54.1)	161 (45.9)				
Type of consultation performed	Not done	159 (31.2)	72 (45.3)	87 (54.7)	1.75 [1.17, 2.61]	.0053		
	Preventiveª	252 (49.4)	149 (59.1)	103 (40.9)	—			
	Treatment	99 (19.4)	41 (41.4)	58 (58.6)	2.05 [1.28, 3.28]	.0015		
Intake of cariogenic foods while watching TV	Nª	163 (31.9)	147 (90.1)	16 (10.0)				
	Y	347 (66.7)	115 (33.1)	232 (66.9)	18.16 [10.34, 31.88]	<.0001	14.75 [8.24, 26.40]	<.0001
Child asks for foods that he or she sees on TV	Nª	145 (28.4)	97 (66.8)	48 (33.3)				
	Y	365 (71.6)	165 (45.2)	200 (54.8)	2.42 [1.62, 3.63]	<.0001		
Parents buy foods requested	Nª	161 (31.6)	105 (65.2)	56 (34.8)				
	Y	349 (68.4)	157 (45.0)	192 (55.0)	2.29 [1.56, 3.38]	<.0001		
Parents buy food that they see on TV	Nª	102 (20.0)	84 (82.4)	18 (17.6)				
	Y	408 (80.0)	178 (43.6)	230 (56.4)	6.03 [3.50, 10.40]	<.0001	3.93 [2.09, 7.37]	<.0001

Abbreviations: CI, confidence interval; dmft + DMFT, total of decayed, missing, and filled primary and permanent teeth; min, minutes; N, not; OR, odds ratio; *p* value, probability value; TV, television; Y, yes.

Category of reference.

**Table 2:** Association of variables with consumption of potentially cariogenic foods while watching TV by Brazilian schoolchildren, 2017

Variable		*n* (%)	Intake of cariogenic foods		OR brute (CI 95%)	*p* value	OR adjusted (CI 95%)	*p* value
			N N (%)	Y N (%)				
Age (year)	>11ª	243 (47.6)	78 (31.8)	165 (68.2)				
	≤11	267 (52.4)	85 (31.3)	182 (68.7)	1.02 [0.70, 1.49]	.9042		
Gender	Female	285 (55.9)	87 (52.3)	198 (47.7)	1.13 [0.78, 1.65]	.5243		
	Maleª	225 (44.1)	75 (50.2)	150 (49.8)				
School	Private	278 (54.5)	86 (30.4)	192 (69.6)	1.12 [0.77, 1.63]	.5520		
	Publicª	232 (45.5)	77 (32.9)	155 (67.1)				
TV time	Morningª	193 (37.1)	67 (34.6)	126 (65.4)				
	Night	317 (61.0)	95 (29.8)	222 (70.2)	1.25 [0.85, 1.83]	.2593		
Time spent watching TV (min)	<90ª	289 (56.7)	116 (40.1)	173 (59.9)				
	>90	221 (43.3)	45 (20.4)	176 (79.6)	2.60 [1.74, 3.89]	<.0001	2.38 [1.57, 3.60]	<.0001
Watch TV commercials	Nª	388 (74.6)	125 (32.2)	263 (67.8)				
	Y	122 (23.5)	36 (29.5)	86 (70.5)	1.14 [0.73, 1.77]	.5762		
Buy food you see on TV	Nª	283 (54.4)	103 (36.8)	177 (63.2)				
	Y	227 (43.6)	57 (25.1)	170 (74.9)	1.74 [1.18, 2.55]	.0051		
Buy soft drinks you see on TV	Nª	455 (87.5)	149 (32.5)	306 (67.5)				
	Y	55 (10.6)	13 (23.6)	42 (76.4)	1.56 [0.81, 2.99]	.1836		
Buy oral hygiene products you see on TV	Nª	491 (94.4)	156 (32.0)	332 (68.0)				
	Y	19 (3.6)	4 (21.0)	15 (79.0)	1.76 [0.58, 5.40]	.3212		
Visit to the dentist	N	159 (31.2)	47 (29.6)	112 (70.4)	1.15 [0.76, 1.72]	.5130		
	Yª	351 (68.8)	115 (32.5)	236 (67.5)				
Type of consultation performed	Not done	159 (31.2)	47 (29.6)	112 (70.4)	—			
	Preventiveª	252 (49.4)	86 (34.4)	166 (65.6)				
	Treatment	99 (19.4)	27 (27.6)	72 (72.4)	21.38 [0.82, 2.31]	.0015		
Child asks for foods that he or she sees on TV	Nª	145 (28.4)	64 (44.4)	81 (55.6)				
	Y	365 (71.6)	97 (26.4)	268 (73.6)	1.38 [0.82, 2.31]	.2208		
Parents buy foods requested	Nª	161 (31.6)	68 (42.1)	93 (57.9)				
	Y	349 (68.4)	94 (26.7)	255 (73.3)	2.12 [1.32, 3.42]	.0019		
Parents buy food that they see on TV	Nª	102 (20.0)	55 (55.6)	44 (44.4)				
	Y	408 (80.0)	105 (25.7)	303 (74.3)	3.46 [1.99, 6.01]	<.0001	3.29 [2.07, 5.24]	<.0001

Abbreviations: CI, confidence interval; min, minutes; N, not; OR, odds ratio; *p* value, probability value; Ref, reference category; TV, television; Y, yes.

Category of reference.

Regarding the control of dental caries, 68.6% of the sample visited the dentist regularly; however, in the last 6 months, approximately 50% of the sample performed preventive procedures (prophylaxis and application of fluoride). Of those who performed some type of procedure (*n* = 99), 95.6% performed restorative treatments and 4.55% performed exodontia.

The 420 hr of recording of the two TV channels (Channel A and Channel B) presented 14,582 commercial advertisements, of which 18.67% (Channel A) and 13.80% (Channel B) were advertisements for food and beverages (Table 3). A higher frequency of food advertisements was shown during the vacation period than during the school period (*p* < .05). The lowest percentages of advertisements were for natural foods and meats. The highest frequencies were for products that were ultraprocessed and that had high‐sugar content.

**Table 3:** TV advertisements according to the content, frequency of presentation, food pyramid group, the type of processing (in natural, culinary ingredients, processed, and ultraprocessed), and the sugar content (Brasil, 2017–2018)

Period	School	Vacation	Sugar content	
	*n* (%)	*n* (%)	g/100 g	*p* value (χ^2^)
In natural foods				
G5				
Meat and poultry	75 (1.58)	122 (2.57)	0.00	.008
Ultraprocessed foods				
G3				
Fruit juice	105 (2.21)	210 (4.43)	13.54	<.0001
G4				
Yoghurt	152 (3.20)	251 (5.29)	7.06	<.0001
Petit suisse cheese	150 (3.16)	210 (4.43)	12.50	.0016
G5				
Sausage	75 (1.58)	150 (3.16)	3.61	<.0001
Ham	78 (1.64)	120 (2.53)	0.00	.0028
G7				
Margarine	112 (2.36)	123 (2.59)	0.10	>.05
Mayonnaise	75 (1.58)	98 (2.06)	10.44	>.05
G8				
Chocolate	90 (1.90)	150 (3.16)	49.22	<.05
Cookie	105 (2.21)	130 (2.74)	36.53	>.05
Sweet candy	0 (0.00)	96 (2.02)	49.00	—
Cola drink	122 (2.57)	210 (4.43)	9.53	<.05
Guarana drink	96 (2.02)	150 (3.16)	10.00	<.05
Chocolate milk	115 (2.42)	180 (3.80)	77.24	.002
Others				
Alcohol (beer)	210 (4.43)	215 (4.53)	NI	>.05
Frozen food	98 (2.06)	132 (2.78)	0.18	.02
Sandwiches (fast food)	18 (2.70)	176 (3.71)	6.30	.0005
Supermarkets	75 (1.58)	152 (3.20)	—	—

Abbreviations: G3, group of fruits; G4, group of milk, yoghurt, and cheese; G5, group of meat, poultry, fish, and eggs; G7, group of fats, spreads, and oils; G8, group of sugars and candy; NI, no informed; *p* value, probability value; χ^2^, chi‐squared test.

## 4 DISCUSSION

The study demonstrated a strong association between the daily time spent by schoolchildren watching TV and outcomes. Schoolchildren who indicated that they watched TV for more than 90 min were more likely to consume cariogenic foods and have a higher rates of caries, which corroborates the findings of other studies showing that greater time spent watching TV is associated with the risk of developing dental caries(Ghimire & Rao, 2013; Zeng, Sheiham, & Sabbah, 2014). Moreover, most participants reported buying or asking their parents to buy products viewed in TV advertisements, with most products referring to food. The marketing of food products has an influence on the construction of inappropriate eating habits in childhood and adolescence by directing their food choices towards foods of low nutritional value, after only a brief exposure to TV commercials(Barr‐Anderson, Larson, Nelson, Neumark‐Sztainer, & Story, 2009; Borzekowski & Robinson, 2001).

The results showed that one third of the commercials analyzed were for food and beverages, most of which were ultraprocessed and had a high‐sugar content, and were exhibited for 14 hr daily and more frequently during school holidays. Healthy food markers, such as fresh foods (meat and poultry), were observed in only 4% of the advertisements, but fruits and vegetables were not observed in the recording period. Similar results were found in a study that analyzed 432 hr of programming in three broadcasters and found that more than 50% of food advertisements concentrated on fats and sweets, with a higher frequency of display at night; an absence of fruit and vegetable advertisements was also found(Almeida, Nascimento, & Quaioti, 2002). Likewise, in another study, a higher frequency of high‐sugar food advertisements was visualized during school vacations, demonstrating the influence of this period on the distribution of advertisements on TV channels(Hawkes, 2004).

The marketing of food and nonalcoholic beverages has been regulated in several countries as one of the strategic measures implemented by the WHO to combat CNCDs, including dental caries(Hawkes, 2004; World Health Organization, 2017). In Brazil, although there are legal instruments that protect children and adolescents against the negative effects of marketing, we still see disagreements between the industrial sector and food advertising regulatory agencies, which lead to noncompliance with established norms(Henriques, Sally, Burlandy, & Beiler, 2012).

Most of the schoolchildren used TV as a form of entertainment, particularly at night, thus being exposed to the commercials broadcast at that time. Audience patterns of children and adolescents have been changing over the years. Now, TVs are present in bedrooms and can be connected to smartphones, tablets, and notebooks, which allow children to watch TV at different times(Morgan et al., 2009). For this reason, the broader recording schedule of the commercials was sought from 8:00 a.m. to 10:00 p.m. uninterrupted, differing from the design of other studies in which the recordings were intervals or only in the morning(Almeida et al., 2002; Vilaro, Barnett, Watson, Merten, & Mathews, 2017). In addition, advertisements for foods considered cariogenic are scarce in children's schedules but are frequent in the intervals of other schedules during which children also watch TV(Al‐Mazyad, Flannigan, Burnside, Higham, & Boyland, 2017).

Compared with parents who were not influenced by TV advertisements, parents who bought food influenced by TV advertisements had children (schoolchildren) who were more likely to consume cariogenic foods in front of the TV and who had higher rates of dental caries. The availability of food for family consumption depends directly on the choices made by parents at the time of purchase, and marketing is an important factor influencing these choices, especially when associating foods with low nutrient content with more favorable health conditions(Dallazen et al., 2018).

The results showed that schoolchildren who consumed a potentially cariogenic diet while watching TV were more likely to develop the disease than those who consumed noncariogenic foods (14 times). An in vitro study demonstrated that the loss of enamel minerals was directly proportional to the frequency of exposure to sucrose and a single daily exposure to 10% sucrose for 5 min induced 20% more demineralization when compared with the control group exposed to 0.9% NaCl. Therefore, there is a positive dose–response relationship between the ingestion of fermentable carbohydrates and dental caries(Diaz‐Garrido, Lozano, & Giacaman, 2016).

Most of the students received professional dental care in the last 6 months, mainly through dental prophylaxis. The presence of the dentist with the majority of schoolchildren who attended dental consultations in the last 6 months did not guarantee the absence of dental caries manifestations; however, it seemed to guarantee disease control, because the mean value of dmft + DMFT was 1.74, below the Brazilian average for the age of 12 years, which was 2.07 in the last epidemiological survey of dental caries disease (SB Brazil 2010).

The research presents some limitations that need to be mentioned. The cross‐sectional study does not establish a causal relationship between the variables and outcomes, but the results indicated that children and adolescents exposed to food and drink advertisements are more likely to develop caries, especially those who consume cariogenic foods in front of the TV and those whose parents, also influenced by the advertisements, usually make these foods available at home. In addition, the food and beverage marketing legislation in force in Brazil is specific and restricted to that country, differing from those in other countries that may present greater rigor in relation to the control of the advertisements on their TV channels.

Dental toothbrushing with fluoridated paste was not addressed in the questionnaire applied, because the quality of the brushing and the fluoride concentration of the pulp used were not evaluated, because it is known that the use of fluorides in an appropriate way can interfere positively in the relation between the consumption of sugar and dental caries, thus reducing the occurrence of this disease(Cury & Tenuta, 2014). However, all the students in the sample had access to fluoridated water supply, which contributes to other findings in which the disease occurred even in the presence of fluoride(Peres et al., 2016). Other factors that can interfere in the occurrence of caries disease, such as the composition of the dental biofilm and saliva and the efficiency of salivary flow and deglutition, were not considered in this study, so we suggest new studies that contemplate these variables.

Improper dietary habits, such as irregular breakfast and a lack of daily consumption of vegetables, are associated with dental caries(Silveira, Prado, Abreu, Serra‐Negra, & Auad, 2018). Therefore, the early consumption of healthy food markers, such as fruits and vegetables, which are sources of essential nutrients in the development of children and adolescents, contributes to the establishment of appropriate eating habits that have a positive impact on health throughout life. Stimulated and reinforced by more populous resources such as TV and through intersectoral actions.

## 5 CONCLUSION

We conclude that children and adolescents aged 10 to 12 years who watch TV for more than 90 min daily are more likely to consume cariogenic foods, which may impact the increase in the rate of dental caries. In addition, parents who buy food and beverages influenced by advertisements contribute to the consumption of cariogenic foods and, therefore, to higher rates of caries in their children.

Moreover, a greater frequency of advertisements for ultraprocessed products with higher sugar content in TV, especially during the vacation period, demonstrates the focus of industries in stimulating the purchase of these products by children and adolescents.

### BULLET POINTS

The present research contributes to the pediatric dentist being more able to evaluate individual risk factors for higher sugar consumption associated with the use of television as entertainment by children and adolescents, which can contribute to the occurrence of dental caries, thus allowing for the elaboration of multiple approaches to the dietary counselling of their patients and therefore for better control of the disease. In addition, this study contributes to sediment population strategies of food education in the fight against caries and other CNCDs that present common determinants.

## CONFLICT OF INTEREST

The authors declare that they have no conflict of interest.

## AUTHOR CONTRIBUTIONS

R. N. M. T. S., D. A. D., and A. M. G. O. conceived the ideas. R. N. M. T. S. collected the data. R. N. M. T. S. and D. A. D. analyzed the data. R. N. M. T. S., D. A. D., and A. M. G. O. led the writing.
